# Development of juvenile sprint performance in boys: analysis of speed phases—a cross-sectional study by age

**DOI:** 10.3389/fspor.2025.1701476

**Published:** 2025-12-05

**Authors:** Simon Bleeker, Maximilian Siener

**Affiliations:** 1 Department of Health Sciences, Medical School Hamburg, Hamburg, Germany; 2 Department of Sport Science, University of Bayreuth, Bayreuth, Germany

**Keywords:** sprint performance, maximum velocity, acceleration phase, adolescent boys, sprint phases

## Abstract

**Introduction:**

Sprinting performance in youth is typically assessed using fixed distances, although sprinting consists of distinct phases that develop differently across age. Little is known about how acceleration, maximum velocity (*V*_max_), and deceleration phases change during growth. This study aimed to analyze sprint phase distribution in boys aged 12–19 years and to develop practical models for estimating key sprint parameters when advanced measurement systems are unavailable.

**Methods:**

A total of 117 boys performed maximal 100 m sprints, with continuous velocity recorded via a 100 Hz laser distance meter. Polynomial–smoothed velocity curves were used to identify acceleration, *V*_max_, and deceleration phases. Differences across age groups (U12–U20) were examined using ANOVA and Tukey–HSD. Multiple linear regression assessed the contribution of each phase to 100 m sprint time. Nonlinear regression models estimated *V*_max_ and acceleration distance based on age and 50 m sprint time.

**Results:**

Acceleration distance increased from 18.8 m (U12) to 24.1 m (U20), whereas deceleration distance declined substantially from 52.4 m to 22.3 m. The *V*_max_ phase more than doubled with age (24.7 m to 47.3 m). Acceleration distance was the only significant predictor of 100 m time (*p* < 0.001). The nonlinear model predicting *V*_max_ demonstrated strong accuracy (*R*² = 0.784), and the model predicting acceleration distance explained 59.8% of the variance.

**Discussion:**

Sprint phase distribution changes markedly during adolescence, with older boys demonstrating longer acceleration and *V*_max_ phases alongside reduced deceleration. Extended acceleration phases are the strongest determinant of 100 m performance. The presented regression tools offer practical options for estimating *V*_max_ and acceleration characteristics when advanced technology is not available.

## 1 Introduction

Sprinting speed is a key determinant of performance in numerous sports, including track and field, soccer, and basketball (1, 2).

Over recent years, the demands on athletes in team sports have increased significantly, with higher sprint distances and frequencies reported during matches. For example, (3) reported an 85% rise in the number of sprints in the English Premier League over seven seasons. This trend is expected to continue, with game speed gaining even greater importance (4). The crucial role of sprinting ability for team-sport performance has been consistently highlighted in several systematic reviews and meta-analyses (1, 2, 5, 6), emphasizing its relevance for repeated-sprint ability and high-intensity match actions in both male and female athletes.

Movement analysis studies have demonstrated that sprinting is a complex, multi-phase motor skill involving coordination, force application, and stiffness control. For instance, comparative kinematic analyses of prepubescent and adult sprinters revealed significant age- and sex-related differences in step parameters and lower-limb joint kinematics, highlighting sprinting as a key indicator of technical and neuromuscular development (7). Similarly, Čoh et al. (8) demonstrated that kinematic and kinetic differences during the sprint start and acceleration phases—particularly in joint coordination and force production—are decisive for sprint performance. These findings emphasize that sprinting is not only a key determinant of athletic performance but also a valuable model for studying movement efficiency and neuromuscular control.

In children and adolescents, sprint performance is a marker of athletic talent (9) and an essential component of motor competence (10) and overall physical fitness (11), which is an important predictor of current and future health outcomes (12). Sprinting tests are widely used in nearly all motor competence assessments (13). Thus, they are particularly relevant as motor competence correlates positively with physical activity and sports participation, while it shows a negative association with high BMI and body fat percentage (14, 15).

Despite the great importance of sprint assessments (16), no standardised test methods have been used to date. For example, in a systematic review of anaerobic capacity development in children and adolescents (17) analyzed studies utilizing 40 m, 50 m, and 50-yard sprints (45.72 m). In contrast, the German Motor Test 6–18 (GMT 6–18) uses a 20 m sprint to assess speed capabilities (18). In soccer talent identification, sprint tests typically range from 10 to 30 m (19, 20) while in basketball, 20 m sprints are performed by players aged 12 years and older (1). In handball, linear sprint performance is commonly assessed over 20 m using light gates to record split times at 5 and 10 m, as shown by Krzysztofik et al. (21). In these studies, speed is often treated as a single physical attribute and evaluated through total sprint times (22). Moreover, standardized sprint distances are typically applied uniformly across all age groups (23).

Nevertheless, the common practice of using predetermined sprint distances across all age groups can measure different aspects of performance depending on age. For example, a 10 m sprint may primarily reflect $V_{\text{max}}$ in younger children, while in adolescents, it may only partially capture acceleration ability. A sprint race has distinct phases, each with unique biomechanical and physiological demands (24). The 100 m sprint is traditionally divided into three main phases: the acceleration phase, the phase of $V_{\text{max}}$, and the deceleration phase (25). Among these, the acceleration phase is particularly critical. This phase is vital for elite sprinters (26) and national-level and juvenile sprinters (27). Elite sprinters accelerate more rapidly and sustain it longer, averaging an additional 11.22 m (35.2%) farther than juvenile sprinters. This capability is equally critical in team sports, where achieving higher velocities over short distances often determines success (28). The $V_{\text{max}}$ phase is defined by a relatively constant velocity, appearing as a flat segment on the velocity-distance curve, where the velocity stays near 100% of the $V_{\text{max}}$ (29). Building on this, the $V_{\text{max}}$ achieved after the acceleration phase strongly correlates with overall sprint performance and distinguishes faster from slower sprinters (30). A higher $V_{\text{max}}$ allows athletes to sustain greater speeds for longer durations, significantly enhancing 100 m sprint performance (30). Additionally, $V_{\text{max}}$ is a key factor for talent identification in team sports (31) and is valuable for programming exercise intensities and monitoring competitive load (32). The deceleration phase in a 100 m sprint is characterized by a gradual decrease in velocity due to fatigue (16). In contrast to the strong correlations observed with $V_{\text{max}}$ and acceleration ability, no significant relationship has been observed between velocity loss and 100 m sprint performance (30).

While these detailed analyses of distinct sprint phases, including $V_{\text{max}}$, have been extensively conducted on elite sprinters (16, 33) or in comparative studies of world-class female sprinters and national-level juvenile sprinters (30), there is still a lack of research focusing on the general population, e.g., students. Although some researchers have addressed acceleration zones for children and adolescents, their definitions are often predetermined and vary considerably, ranging from 10 m (34) to 20–30 m (22). Overall, numerous studies have attempted to quantify sprinting ability in children. However, a comprehensive analysis of the importance of sprint phases for sprint time, especially in adolescence, is missing. In addition, the influence of $V_{\text{max}}$ on sprint performance in boys during growth has not yet been fully clarified. Furthermore, many coaches do not have access to advanced measurement tools. This can pose challenges when assessing key sprint aspects such as acceleration ability, $V_{\text{max}}$, and deceleration in children and adolescents.

Therefore, this study aims to provide a detailed analysis of sprinting abilities in children and adolescents. It will establish distinct sprint phases, including the acceleration phase, the $V_{\text{max}}$ phase, and the deceleration phase, for different age groups. In doing so, it seeks to offer more comparable and meaningful insights into the progression of sprinting performance in boys during growth. Furthermore, this study aims to develop regression-based formulas to predict $V_{\text{max}}$ and distance to $V_{\text{max}}$ using absolute sprint times and age. These tools can support coaches and practitioners who lack access to advanced technologies such as photoelectric cells or laser measurement devices.

Based on these aims and the existing literature, two hypotheses were formulated: (1) The distribution of sprint phases changes with age, with older boys exhibiting longer acceleration and $V_{\text{max}}$ phases. (2) Among the distinct sprint phases, acceleration distance is the primary predictor of 100-m sprint time in adolescent boys, whereas ($V_{\text{max}}$) and deceleration distance show no significant contributions.

## 2 Materials and methods

### 2.1 Study design

This study employed a cross-sectional design to analyze sprint performance in boys across different age groups (U12–U20). Therefore, each participant performed a maximum-effort 100 m sprint. Sprint phases—acceleration, $V_{\text{max}}$, and deceleration—and performance differences between the age groups were examined. Additionally, models were developed to predict $V_{\text{max}}$ and distance to $V_{\text{max}}$ based on age and 50 m sprint times.

### 2.2 Participants

Overall, 117 boys aged 12 to 19 years (14.57 ± 2.20 years) participated in the study. All participants were enrolled in grades 5 to 12 and reported no known medical conditions that could affect their performance. Participants were recruited from local schools (Germany). The cohort consisted of males, divided into five age groups: U12 (10–11 years), U14 (12–13 years), U16 (14–15 years), U18 (16–17 years), and U20 (18–19 years). Age group classifications in two-year intervals, such as U12, U14, and U16, are commonly applied in sports like Handball (35), Soccer (36), and Basketball (37). Furthermore, these age groups account for developmental differences, supported by research indicating significant performance changes approximately every two years (34). To minimize variability, all sprint tests were conducted on a synthetic track under consistent environmental conditions. An a priori sample size calculation for a multiple regression analysis with two predictors was conducted. Assuming a large effect size (f2 = 0.35), a significance level of α = 0.05, and a desired power of 0.8, the required sample size was determined to be 31 participants. The sample size exceeded this requirement, ensuring sufficient statistical power for the analysis. Ethical approval for this study was obtained from the Ethics Committee of the University of Bayreuth (Approval No.: Az. O 1305/1 – GB: No. 25-006) Informed written consent was obtained from both the participants and their legal guardians. Participation in the study was voluntary.

### 2.3 Tests/procedures/measurements

The warm-up was conducted according to established protocols (38) and supervised by a professional to ensure safety and consistency. The sprint trials began from a standing position. Each sprint was initiated with a countdown of “3, 2, 1, Go.” Upon the “Go” command, participants sprinted at maximum effort beyond the 100 m mark before slowing down. Simultaneously, the start button on the measuring device was activated. Participants were instructed to maintain a stable standing position during the countdown and to exert maximum effort during the sprint. They were instructed on running through the finish line to ensure consistent effort across trials. All sprint trials were conducted on a standard synthetic outdoor track surface, and testing took place in the morning (10:00–12:00 a.m.) to reduce potential effects of circadian variation. To ensure reliability, each participant performed two maximal 100 m sprints with 5 min recovery, and the faster trial was used for analysis to represent their best performance (39). Sprint performance data were collected using a laser distance meter (LDM-300 C, Jenoptik, Jena, Germany). The device measures speed with an accuracy of ±0.1 m/s at a sampling rate of 100 Hz. Harrison et al. (40) reported high reliability for a laser-based system (ICC = 0.986) compared to video cameras at 50 Hz (ICC = 0.984) and 100 Hz (ICC = 0.981) in measuring velocity. Displacement errors were comparable across systems, highlighting the need for appropriate filtering to ensure accurate velocity. These findings confirm the validity of the LAVEG system for capturing sprint velocity and displacement in field settings. Previous studies have reported high test–retest reliability for sprint times up to 100 m (ICC = 0.986) (41), supporting its use as a standardized and reproducible field test in youth populations.

The device was positioned approximately 1 m behind the participants and corrected for perspective error using Doppler device sampling software (DAS3E v3.9, Jenoptik Laser, Jena, Germany). The laser beam targeted participants’ backs at a height of 1 m above the ground, with the horizontality of the beam carefully maintained to ensure accurate measurements (42). Age and gender were entered into the software, automatically correcting for perspective errors using the Pythagorean theorem. Initially, the spatial data were smoothed using a zero-lag moving average filter with a window size of 50 ms, applied within the Doppler device sampling software (DAS3E v3.9, Jenoptik Laser, Jena, Germany). The spatial data collected with the LAVEG system were further processed and analyzed using MATLAB (R2024b, MathWorks®, Natick, Massachusetts, USA). A 4th-order Butterworth low-pass filter with a cut-off frequency of 0.8 Hz was then applied to reduce high-frequency noise (43). The filtered data were converted to instantaneous velocity measurements using the first central difference equation as shown in Equation 1.

$$v_{i}=\frac{\left(X_{i+1}-X_{i-1}\right)}{2t},$$ (1)

where v is the velocity, i is the frame of interest, and t is the time interval between data points (0.01 s). These instantaneous velocity measurements were then trimmed to the relevant sprint segment, with the sprint start defined as the first point where velocity exceeded 0.2 m/s (42). To approximate the trimmed velocity data, a 5th-degree polynomial was fitted using least-squares regression (44). This method was applied to reduce fluctuations and inherent noise within the data (45). For the velocity analysis, phases were defined as follows: the acceleration phase extended until acceleration fell below 0.1 ${m/s}^{2}$ (46). The $V_{\text{max}}$ phase began at the end of the acceleration phase and continued until velocity fell below 95% of the $V_{\text{max}}$ (29). Finally, the deceleration phase followed the $V_{\text{max}}$ phase. Distances covered during each phase were calculated using trapezoidal integration. For each participant, the distance covered in each phase was extracted and analyzed. Subsequently, the mean distances for each phase were computed across all participants within each age group to facilitate group-level comparisons.

### 2.4 Statistical analysis

All statistical analyses were performed using R (Version 4.3.2; R Core Team, 2024). Significance level (two-sided) was set at α=0.05. Pairwise comparisons were made in all calculations, and missing values were excluded by default. Normality of the data was examined within each age group (U12–U20) using the Kolmogorov–Smirnov test (47). All sprint time (10–100 m) and velocity variables were normally distributed (p<0.05).

Descriptive analyses of participants’ sprint performances were conducted across various distances (10, 20, 30, 40, 50, and 100 m) and for $V_{\text{max}}$.

Descriptive statistics, including mean, standard deviation, minimum, and maximum values, were calculated for each variable within each age group. An analysis of variance (ANOVA) was performed to determine significant differences in sprint performance across the age groups. When ANOVA results were significant, Tukey’s Honest Significant Difference (HSD) test was applied post hoc to identify specific group differences. Following the post hoc tests, Cohen’s d was calculated to quantitatively assess the effect sizes of the significant differences (48, 49).

To test Hypothesis 2, a multiple linear regression analysis was conducted to evaluate how distances covered during the sprint phases influenced the time to complete 100 m. The independent variables included distances covered during the acceleration, $V_{\text{max}}$, and deceleration phases.

A separate nonlinear regression model was developed to estimate the distance to $V_{\text{max}}$ based on the 50 m sprint time and athlete age.

Furthermore a nonlinear regression model was developed to predict $V_{\text{max}}$ from 50 m sprint time and age, with both predictors entered simultaneously. The nonlinear regression model was fitted, with initial parameter estimates provided to ensure convergence.

## 3 Results

The ANOVA results revealed significant differences across age groups for most sprint variables, including 20, 30, 40, 50, 100 m, and $V_{\text{max}}$ (p<0.001), with all corresponding results presented in Table 1. Post hoc Tukey-HSD tests, limited to adjacent age groups, showed no significant difference between U12 and U14 (p=0.999,d=−0.07). A significant improvement was observed between U14 and U16 (p<0.001). Cohen’s d values (min/max: 1.086/1.776) indicated large to very large effects. Significant differences were also found between U16 and U18 in the 50 m, 100 m, and $V_{\text{max}}$ variables (min/max: p=0.026/0.003, d=−0.70/0.78), highlighting moderate to large effects. No significant differences were found between U18 and U20 (p=0.999,d=0.09).

**Table 1:** Sprint performance metrics across age groups.

Age group	U12 (10.9 ± 0.3 y; *n* = 13)	U14 (12.6 ± 0.5 y; *n* = 35)	U16 (14.5 ± 0.5 y; *n* = 28)	U18 (16.5 ± 0.5 y; *n* = 29)	U20 (18.2 ± 0.4 y; *n* = 12)
10 m	2.43 ± 0.19	2.48 ± 0.18	2.17 ± 0.16	2.17 ± 0.14	2.15 ± 0.07
Min/Max	2.13/2.74	2.17/2.94	1.76/2.48	1.93/2.48	2.02/2.24
Cohen’s *d*		*d* = 1.33		*d* = 0.20	
20 m	4.08 ± 0.29	4.08 ± 0.34	3.59 ± 0.23	3.52 ± 0.20	3.45 ± 0.11
Min/Max	3.68/4.62	3.58/4.91	3.16/4.11	3.19/3.96	3.29/3.62
Cohen’s *d*		*d* = 1.27		*d* = 0.17	
30 m	5.70 ± 0.41	5.63 ± 0.52	4.96 ± 0.32	4.81 ± 0.27	4.68 ± 0.17
Min/Max	5.13/6.46	4.92/6.91	4.33/5.68	4.38/5.41	4.43/4.96
Cohen’s *d*		*d* = 1.78		*d* = −0.32	
40 m	7.37 ± 0.54	7.23 ± 0.73	6.34 ± 0.44	6.10 ± 0.35	5.90 ± 0.25
Min/Max	6.61/8.38	6.28/9.05	5.51/7.33	5.58/6.91	5.53/6.31
Cohen’s *d*		*d* = 1.70		*d* = −0.48	
50 m	9.10 ± 0.68	8.89 ± 0.96	7.76 ± 0.57	7.41 ± 0.44	7.15 ± 0.34
Min/Max	8.10/10.37	7.66/11.29	6.67/9.07	6.79/8.49	6.63/7.69
Cohen’s *d*		*d* = 1.63		*d* = −0.57	
100 m	17.44 ± 1.31	17.33 ± 2.09	14.87 ± 1.25	14.08 ± 1.00	13.57 ± 0.81
Min/Max	15.50/19.90	14.40/22.60	12.50/17.60	12.80/16.60	12.30/14.70
Cohen’s *d*		*d* = 1.52		*d* = −0.70	
** $V_{\text{max}}$ **	22.55 ± 1.66	23.64 ± 2.55	26.70 ± 2.14	28.37 ± 1.76	29.70 ± 1.83
Min/Max	19.70/25.15	18.44/27.54	22.58/31.19	25.04/31.94	26.98/32.90
Cohen’s *d*		*d* = 1.09		*d* = 0.78	

Values in parentheses indicate mean chronological age (years) ± SD and sample size per group. All values are presented as mean ± SD. Min/Max values are shown below each row. Distances are measured in meters (m); $V_{\text{max}}$ is presented in km/h. Cohen’s *d* represents effect sizes between U14–U16 and U16–U18.

### 3.1 Sprint phases

**Figure 1:**
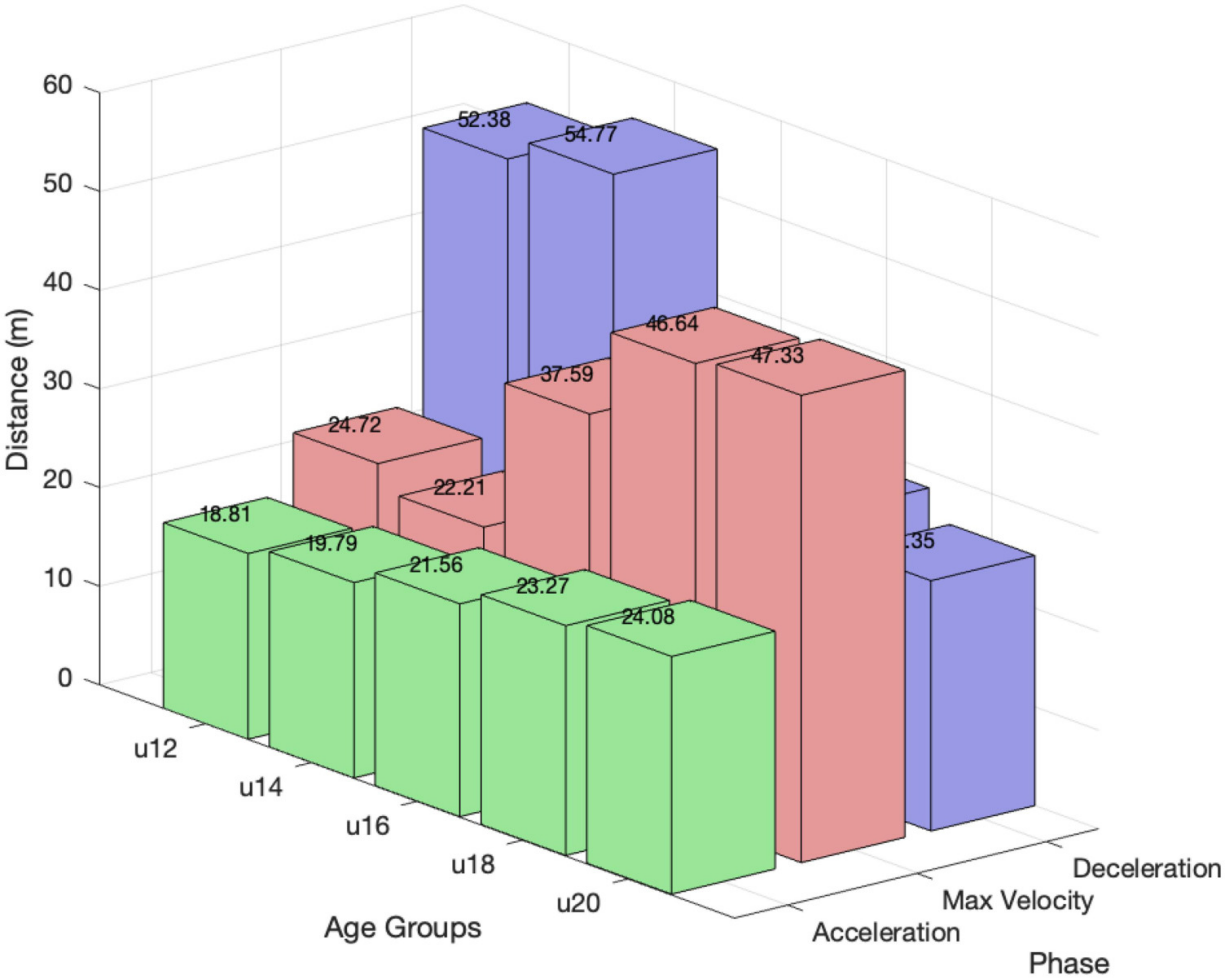
Three-dimensional bar chart illustrating mean sprint phase distances for the acceleration (green), $V_{\text{max}}$ (red), and deceleration (blue) phases across age groups (U12–U20). Values on each bar represent group means (in meters). The chart highlights the age-related increase in acceleration and $V_{\text{max}}$ distances and the pronounced reduction in deceleration distance in older age groups.

The analysis revealed apparent quantitative differences in the average distances covered across the sprint phases and age groups. The mean acceleration distance increased with age, from 18.8 m (±1.11) in the U12 group (10–11 years) to 24.1 m (±3.27) in the U20 group (18–19 years), representing an increase of approximately 28%. In contrast, the deceleration phase showed a substantial decrease with age, with, for example, the U14 athletes covering an average of 54.8 m (±11.5) compared to only 22.3 m (±11.7) in the U20 group (18–19 years), representing a reduction of approximately 60%. The $V_{\text{max}}$ phase exhibited the opposite trend, with older athletes covering significantly greater distances. The U14 group achieved a mean distance of 22.2 m (±11.1), while the U20 group reached 47.3 m (±12.5). This age-related pattern in sprint phase distribution is illustrated in Figure 1.

**Figure 2:**
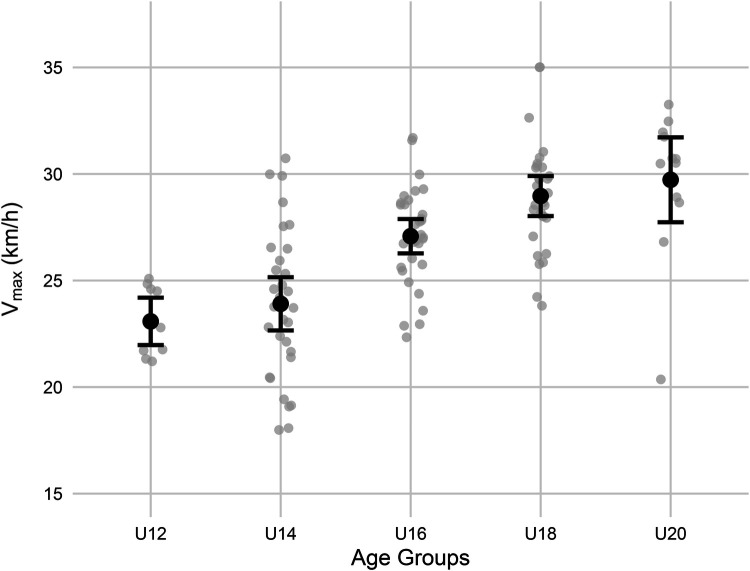
$V_{\text{max}}$ across age groups (U12–U20) in boys. Each black dot indicates the group mean with 95% confidence intervals, and gray dots represent individual participants. The figure demonstrates a clear age-related increase in $V_{\text{max}}$, with greater between-subject variability in older age groups.

Supporting Hypothesis 2, the regression analysis revealed that acceleration distance was a significant negative predictor of 100 m sprint time (β=−0.411, 95% confidence interval (CI) [−0.488,−0.334], p<0.001. In contrast, the $V_{\text{max}}$ phase (β=−0.011, 95% CI [−0.069,0.046], p=0.695; Figure 2 illustrates the age-related progression and variability in maximum velocity) and the deceleration phase (β=0.026, 95% CI [−0.031,0.083], p=0.369) did not significantly predict sprint time. The model explained 69.5% of the variance in 100 m sprint time ($R^{2}=0.695$, adjusted $R^{2}=0.690$), with a standard error of estimate (SEE) of 1.16 s.

A nonlinear regression model was applied to predict the distance required to reach maximum speed (*Distance Acceleration Phase*) based on the 50 m sprint time and athlete age. The equation of the model is:

$$\text{Distance\_Acceleration\_Phase}=180.0\cdot {\text{Time\_to\_50 m}}^{-1.07}+0.11\cdot \text{Age}$$ (2)

**Figure 3:**
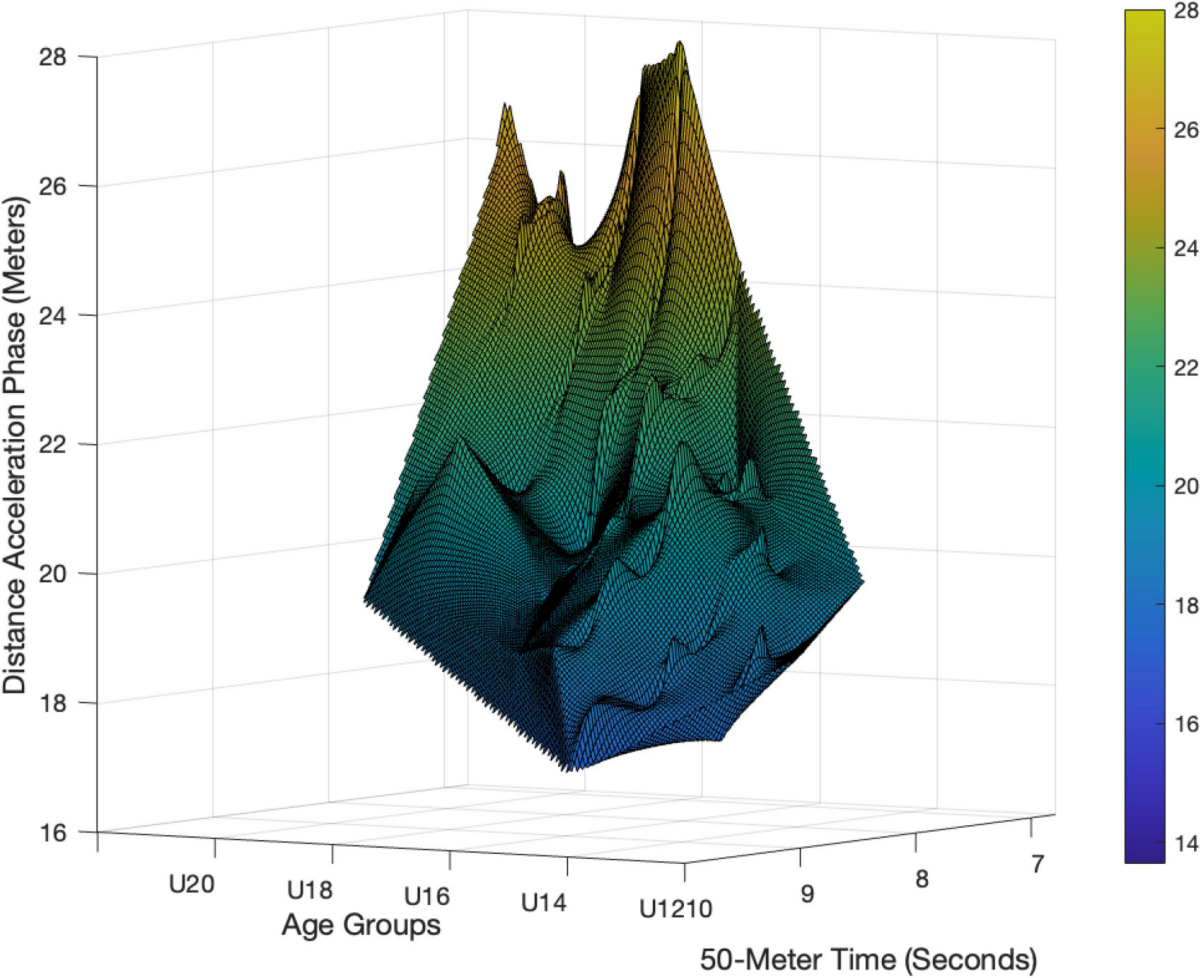
Three-dimensional surface plot showing the modeled relationship between the acceleration distance (*y*-axis), 50 m sprint time (*x*-axis), and age group (*z*-axis) in boys. Color shading represents acceleration distance (m). The surface illustrates a nonlinear interaction between sprint time, age, and acceleration distance, with a curved gradient across both performance and age dimensions.

The model explained 59.8% of the variance in the acceleration distance ($R^{2}=0.598$, adjusted $R^{2}=0.591$), with a standard error of estimate (SEE) of 1.97 m. The coefficient for 50 m sprint time was estimated at −1.07 (95% CI [−1.22,−0.93], p<0.001), indicating that longer sprint times were associated with shorter acceleration distances. The coefficient for age was estimated at 0.11 (95% CI [−0.05,0.27], p=0.176), showing that age did not significantly contribute to the prediction of acceleration distance. The scaling constant of the model was 180.0 (95% CI [134.0,240.0]) as presented in Equation 2. The modeled relationship between 50 m sprint time, age, and acceleration distance is visualized in Figure 3.

### 3.2 Maximum velocity

Furthermore, a nonlinear regression model was developed to predict $V_{\text{max}}$ from 50 m sprint time and age, with both predictors entered simultaneously. The nonlinear model was fitted using initial parameter estimates to ensure convergence, resulting in the following equation:

$$V_{\text{max}}=237.0\cdot {\text{Time\_to\_50 m}}^{-1.11}+0.167\cdot \text{Age}$$ (3)

The model explained 78.4% of the variance in $V_{\text{max}}$ ($R^{2}=0.784$, adjusted $R^{2}=0.780$), with a standard error of estimate (SEE) of 1.61 km/h. The coefficient for 50 m sprint time was estimated at −1.11 (95% CI [−1.21,−1.02], p<0.001), indicating that longer sprint times were associated with lower $V_{\text{max}}$. The coefficient for age was estimated at 0.167 (95% CI [0.036,0.298], p=0.013), suggesting that older athletes reached higher maximum velocities. The scaling constant of the model was 237.0 (95% CI [195.0,288.0]) as presented in Equation 3.

## 4 Discussion

Across all age groups, velocity development consistently followed the same pattern, with distinct phases of acceleration, a plateau at $V_{\text{max}}$, and subsequent deceleration. However, we identified apparent age-related differences in the sprint phases. Older athletes demonstrated longer acceleration phases, greater $V_{\text{max}}$ distances, and reduced reliance on deceleration compared to younger children.

These descriptive findings address Hypothesis 1, showing that the distribution of sprint phases changes with age: older boys demonstrated longer acceleration and $V_{\text{max}}$ phases. In U12 boys, the acceleration phase was markedly shorter and the deceleration phase substantially longer. Consequently, during a 70 m sprint, almost two-thirds of the distance would be spent decelerating, whereas in adolescents the same distance is predominantly covered within the acceleration and $V_{\text{max}}$ phases. Thus, shorter sprint distances, such as a 20 m sprint times may not fully capture the sprinting abilities of older children and adolescents (18). This underscores the need for age-appropriate and ability-specific assessments of sprint performance.

These changes may reflect growth-related increases in strength and power as well as improvements in mechanical efficiency. Biomechanical comparisons between adult and prepubescent sprinters indicate that relative step length differences reflect disparities in force-generation capacity. Chatzilazaridis et al. (7) showed that younger athletes exhibit reduced ankle joint contribution during touchdown, resulting in prolonged ground contact times and less efficient horizontal force application. Similarly, Rossi et al. (50) found that adolescents outperform children in 30 m sprints due to higher theoretical maximal velocity ($V_{0}$) and maximal power output ($P_{\text{max}}$), despite comparable theoretical maximal horizontal force per unit body mass ($F_{0}$). This suggests that improved sprint performance with age is primarily a result of more effective force application and coordination, leading to longer relative step lengths and may help explain longer acceleration and $V_{\text{max}}$ phases.

While no studies have specifically analyzed acceleration distances in boys during growth, existing research provides general insights into acceleration phases. For instance, (32) highlighted 5 m sprints as a measure of acceleration capacity due to their logical validity in soccer contexts. Similarly, (34) defined the first 10 m of a 30 m sprint as the acceleration phase. However, these findings differ significantly from the results of our study, as the distances reported in these studies are substantially shorter.

In contrast, (27) documented acceleration distances of up to 32 m for 14-year-old female athletes competing at national championships. This is considerably longer than the distances observed in our sample. This discrepancy can be attributed to differences in the participants’ athletic profiles. Our sample consisted of normal school children, whereas (27) conducted their study on highly trained female athletes. Notably, these athletes also demonstrated a $V_{\text{max}}$ phase of 40 m, which is shorter than the $V_{\text{max}}$ phase observed in the U18 and U20 groups in our study. Therefore, the length of the $V_{\text{max}}$ phase may not necessarily correlate with performance levels. The $V_{\text{max}}$ phase is typically shorter among elite-level sprinters as the acceleration phase becomes more pronounced.

Consequently, our findings also showed that acceleration distance was a significant predictor of 100 m performance, which is consistent with the results of other studies on elite sprinters (26). Faster sprinters tend to exhibit longer acceleration distances than slower sprinters. These results are in line with the conclusion from (27): “the better the final time, the longer the distance of positive acceleration.” Data from elite athletes support this observation. Elite women accelerate up to 46.2 m (10.90 s), while elite men achieve even greater distances of 50.0 m (10.10 s) and 52.1 m (9.90 s). Furthermore, the deceleration phase was not a significant predictor of performance—a result that is in line with previous studies on world-class sprinters (30). While the $V_{\text{max}}$ distance does not appear to play a significant role in predicting 100 m performance among young untrained boys, Usain Bolt’s exceptional success has been attributed to his ability to maintain $V_{\text{max}}$ over an extended phase of 50–80 m (51). This contrast underscores the differing factors that influence sprint performance across varying expertise and development levels.

Marked age-related differences in sprint performance were observed, with the greatest improvements emerging during mid-adolescence. This pattern coincides with the period of rapid growth and neuromuscular adaptation typically occurring around peak height velocity (PHV), emphasizing the strong link between biological maturation and sprint development (52). These findings align with previous research reporting accelerated sprint improvements around puberty. Baker et al. (53) demonstrated moderate-to-large effect sizes favoring post-PHV athletes across 10–30 m sprint distances, while Ruf et al. (54) established normative reference centiles in elite youth soccer players, showing that sprint ability aligns more closely with skeletal rather than chronological age. Similarly, Viru et al. (55) highlighted that motor abilities develop most rapidly between 13 and 16 years, reinforcing the critical influence of maturation on neuromuscular performance. In support of this, Meyers et al. (56) reported that maximal sprinting speed tends to develop at a quicker rate following the onset of the growth spurt, driven by increases in stride length and stabilization of stride frequency and ground contact times. These growth-related changes may partly explain the improved performance observed in older athletes.

Increases in strength and power likely contribute to these age-related improvements in sprint performance (33, 57, 58). Both become increasingly critical as sprint distances shorten, since they determine acceleration capacity (59). Significant differences in strength and muscle size emerge during the later stages of puberty. Gillen et al. (60) reported that post-pubertal males exhibit a 130% greater strength and a 101% larger cross-sectional muscle area compared to pre-pubertal males. Furthermore, metabolic factors contribute to the improvements in sprinting abilities observed in adolescent boys (61). These factors heavily influence sprinting, as it is a maximal anaerobic performance (62). Between the ages of 12 and 17, boys experience greater relative improvements in anaerobic than aerobic fitness (63). During growth and maturation, anaerobic performance improvements are largely explained by increases in muscle mass, glycolytic enzyme activity, and neuromuscular coordination, whereas aerobic adaptations such as enhanced oxidative capacity and cardiac function emerge earlier in development (63). These developments are further reinforced by physical characteristics such as height and lower-limb stiffness. Greater height may enhance stride length and thereby help maintain higher speeds.(64). This may explain the pronounced improvements in $V_{\text{max}}$ observed during adolescence, when growth spurts are common (65). Lower-limb stiffness also plays a key role by facilitating energy transfer and improving efficiency throughout the stride cycle (66).

### 4.1 Future research and limitations

Future research should focus on further investigation of sprint performance in a more diverse population, including girls and athletes participating in different sports with substantial variability in training backgrounds, to strengthen the generalization of the findings. Longitudinal studies could shed more light on how sprint performance progresses over time. In that case, more causal relationships could be established. Furthermore, by incorporating experimental methodologies of more advanced biomechanical analyses such as 3D motion capture, findings could provide additional insights into how alterations in mechanics accompany changes in sprinting during growth and maturation. A limitation of the present study is that no direct measures of biological maturation or detailed anthropometric characteristics were collected. Consequently, the observed differences between age groups reflect chronological rather than biological developmental stages. Future research integrating such parameters may contribute to the creation of functional predictive tools that support coaches and practitioners in optimizing training programs across developmental stages.

This study has laid the groundwork for understanding sprint performance in adolescent boys and provides practical implications for athletic training and testing. Addressing these limitations in future research will help refine our understanding and further improve training methods for young athletes.

## 5 Conclusion

The findings of this study provide clear, hypothesis-driven guidance for coaches and practitioners. First, consistent with our initial assumptions on sprint phase development, test distances should be adapted to the athlete’s age and the specific quality under assessment. For younger athletes (U12), shorter sprints (15–20 m) are most valid for assessing acceleration, whereas older adolescents (U18–U20) require longer test distances (20–30 m) to reflect their extended acceleration phases. If the goal is to capture $V_{\text{max}}$, test distances must be long enough for athletes to reach and briefly maintain their $V_{\text{max}}$, which occurs later during the sprint in older athletes.

Second, in line with our regression models, $V_{\text{max}}$ can be estimated reliably from a 50 m sprint test and the athlete’s age, even without advanced technology such as laser systems or timing gates. This provides a practical tool for performance monitoring and age-appropriate program design.

Our study offers valuable information on the development of sprinting performance across ages, providing a detailed analysis of speed capabilities in children and adolescents. However, restricting the population to adolescent boys from a narrow geographic location may limit the generalizability of results to other populations. Nevertheless, the findings are consistent with previous research (67), supporting the broader applicability of the data. Furthermore, the cross-sectional design of our study precludes the assessment of individual progress over time and the inference of causal relationships between age and sprinting phases.

## Data Availability

The raw data supporting the conclusions of this article will be made available by the authors, without undue reservation.
